# The Effect of Acute Exercise and Psychosocial Stress on Fine Motor Skills and Testosterone Concentration in the Saliva of High School Students

**DOI:** 10.1371/journal.pone.0092953

**Published:** 2014-03-24

**Authors:** Mirko Wegner, Johan M. Koedijker, Henning Budde

**Affiliations:** 1 Institute of Sport Science, University of Bern, Bern, Switzerland; 2 Faculty of Human Sciences, Medical School Hamburg, Hamburg, Germany; 3 Department of Sport Science, School of Science and Engineering, Reykjavik University, Reykjavik, Iceland; Emory University School Of Medicine, United States of America

## Abstract

Little is known about the influence of different stressors on fine motor skills, the concentration of testosterone (T), and their interaction in adolescents. Therefore, 62 high school students aged 14–15 years were randomly assigned to two experimental groups (exercise, psychosocial stress) and a control group. Exercise stress was induced at 65–75% of the maximum heart rate by running for 15 minutes (*n* = 24). Psychosocial stress was generated by an intelligence test (HAWIK-IV), which was uncontrollable and characterized by social-evaluative-threat to the students (*n* = 21). The control group followed was part of a regular school lesson with the same duration (*n* = 28). Saliva was collected after a normal school lesson (pre-test) as well as after the intervention/control period (post-test) and was analyzed for testosterone. Fine motor skills were assessed pre- and post-intervention using a manual dexterity test (Flower Trail) from the Movement Assessment Battery for Children-2. A repeated measure ANCOVA including gender as a covariate revealed a significant group by test interaction, indicating an increase in manual dexterity only for the psychosocial stress group. Correlation analysis of all students shows that the change of testosterone from pre- to post-test was directly linked (*r* = −.31, *p* = .01) to the changes in manual dexterity performance. Participants showing high increases in testosterone from pre- to post-test made fewer mistakes in the fine motor skills task. Findings suggest that manual dexterity increases when psychosocial stress is induced and that improvement of manual dexterity performance corresponds with the increase of testosterone.

## Introduction

There is little relevant research on the effects of different stressors, such as exercise or psychosocial stress, on motor skills [1]. Effects differ regarding the characteristics of the stress intervention (i.e., intensity, duration, and type) and the motor test utilized (e.g., fine or gross motor performance and the time of administration). It has long been known that exercise induced fatigue has a negative effect on movement execution [2], [3], [4], [5]. For example, soccer and basketball passing accuracy has been observed to decrease following a bout of high-intensity, lower-body resistance training [6], [7]. Further, McMorris et al. [8] investigated simple reaction time performances at rest and following cycle ergometry at moderate (70%) and high (100%) percentages of maximal power output and reported that moderate-intensity exercise yielded improvements in reaction times while reaction times increased after heavy exercise. Taken together, the empirical evidence suggests an inverted-U function between exercise intensity and motor control. Regarding whole body exercise, moderate intensity seems to improve performance. However, there is still a lack of research on the underlying mechanisms of the effects of exercise on fine motor skills, such as the possible influence of neurobiological mediators.

Regarding the effects of psychological stress on motor skills most publications found a negative effect of high levels of anxiety on gross motor performance [9], [10], [11], [12], [13], [14]. For fine motor skills, it has been reported in surgery that stress impairs surgical performance while being exposed to theatre background noise at 80 to 85 dB or due to the speed of performance (as quickly as possible) [15], [16]. However, it was also found that motor performance increases under moderate stress conditions [17]. So, similar to the effects of exercise, motor performance can be assumed to follow an inverted-U as a function of the intensity of psychological stress [14]. However, all these studies did not distinguish between psychological and physiological stress and usually did not consider possible neurobiological explanations for the effect on performance.

Typically, research investigating the relationship between different stressors and motor performance attributes the decline in performance primarily to physiological factors (e.g., blood lactate concentrations, insufficient energy resources, dehydration) [2], [3], [4], [5] or psychological factors (overloading of attentional resources, distraction from relevant information sources, disruption of movement automaticity by attending to motor execution) [9], [10], [11], [12], [13]. Other possible mechanisms that may contribute to the association between stress and altered performance are neurobiological but are limited to the effects on cognition and are reviewed by McMorris and Hale [18]. However, none of these reviewed studies measured if the reported changes are due to changes in the release of hormones from the hypothalamic-pituitary-gonadal (HPG) axis [19]. Hormone releases of the HPG axis (like testosterone) could possibly explain the variance in fine motor performance immediately following acute stress bouts. It could be assumed that an androgenic steroid like testosterone exerts affects in the nigrostriatal dopaminergic (NSDA) system [20] by ameliorating the expression of tyrosine hydroxylase (TH) and dopamine transporter (DAT) and consequently improves motor control [21], [22], [23].

Recent animal research shows that testosterone affects motor behavior and the NSDA system in male rats and that exogenous testosterone intervention to aged male rats could ameliorate age-related motor impairment [24]. This is in line with human findings in which men with low testosterone levels caused by androgen deprivation therapy showed worse fine motor skills [25]. Additionally there are results suggesting that the development of general perceptual motor skills are related to participants' testosterone concentration (especially between 12 and 14 years) [26], [27], [28].

Interestingly, none of this research manipulates the testosterone concentration via acute stress. A change in testosterone is easy to induce with stress. Similar to adults, testosterone concentration in adolescents increases in response to a 12-min exercise bout of 70–85% HR_max_ [29], [30]. Related to psychological stress, increased levels of testosterone have been observed in relation to competitions [31], [32], [33] and in response to acute psychosocial stress in both men and women [34].

To our knowledge, no study exists which investigated the influence of different stressors on testosterone concentration and fine motor skills in adolescents. Therefore, as a preliminary effort, the aim of the present study was to analyze whether changes in fine motor performances due to acute physical and psychological stress could be related to changes in testosterone levels. A school setting is often associated with stress and it is important to determine the influence of different school stressors on testosterone because of their effects on various school-related requirements like cognition, motor functioning, and non-aggressive behavior. With regard to the current literature, we hypothesized an increase in testosterone after an exercise intervention (a situation causing physical stress) as well as after a situation producing psychosocial stress. Additionally, we hypothesized that fine motor performance increases in response to moderate exercise and moderate psychosocial stress.

We chose two stressors that were assumed to moderately but significantly change testosterone levels (i.e., exercise at 65–75% of the maximum heart rate, HR_max_; socio-evaluative psychological stressor). To implement the intervention in a school setting we chose a duration of 15 minutes, which has previously been shown to affect steroid hormones in school children and adolescents [29], [30], [35], [36].

## Materials and Methods

### Ethics statement

All participants and their parents signed an informed consent form prior to participation. The present study was approved by the relevant ethics committee of the German Psychological Society (DGPs). The study was performed in accordance with the declaration of Helsinki.

### Participants

62 healthy right-handed high school students from the 9^th^ grade of a Berlin (Germany) school aged 14–15 years participated in this study. The students were randomly assigned to one of two experimental (EG 1: 65–75% HR_max_, EG 2: psychosocial stress) or a control group (CG). An experimenter, who was not further involved in the study, generated the allocation sequence, enrolled, and randomly assigned participants to their groups. Exclusion criteria for study participation were: dyslexia (verified by teachers' statements), obesity, mental or physical impairments, and a history of psychoactive substances (e.g. Ritalin). No participant had to be excluded due to these criteria. The overall sample (*N* = 62; 28 male and 34 female) had a mean age of 14.8 years (*SD* = 0.49). The mean age in the CG was 14.9 years (*SD*  = 0.55, *n* = 23, 11 female), in the EG 1 it was 14.7 years (*SD*  = 0.48, *n* = 20, 11 female), and in the EG 2 it was 14.8 years (*SD*  = 0.41, *n* = 19, 12 female).

### Exercise testing and intervention

#### Maximum performance exercise

This was done as described previously by Budde et al. [30]. The maximum performance in the Shuttle Run Test [37] was used to determine the individual target HR for the exercise intervention. It was performed on a 20-m track. The exercise test started at a speed of 8.5 km/h. Every one minute running speed was increased by 0.5 km/h until exhaustion. At each speed level participants received continuous acoustic signals in a given frequency as pacing signals. We determined the maximum HR achieved at the end of the test. For cardiovascular assessment and to control the exercise intensity we used heart rate monitors (HRM RS400, Polar, Kempele, Finland). The HRM consists of an elasticized chest belt and a computer watch that detects and stores the subject's mean heart rate every 5 s.

#### Exercise interventions

The individual target HR was based on the results of the initial maximum exercise performance (The Shuttle Run Test) [37]. In the exercise group (EG 1), subjects ran in a sport gym on a 60-m track at their individual exercise intensity level (65–75%) based on their individual HR_max_ for 15 minutes. Each subject received an acoustic signal through the heart rate monitor when he or she was running too fast (fast beeping) or too slow (slow beeping). The 15-minute period was chosen because it could be easily integrated as an active exercise break between two classes in a school timetable and has been shown to significantly change testosterone levels [30].

### Psychosocial stressor

The EG 2 group received an intelligence test HAWIK-IV [38] to induce psychosocial stress. The HAWIK-IV includes different mathematical and verbal tests, which have been previously shown to raise stress levels [39], [40]. The testing also lasted for 15 minutes in total. In accordance with Dickerson and Kemeny [41] we designed our task to elicit a stress response due to an uncontrollable situation which was characterized by social-evaluative threat. Therefore, the students were told that their IQ score would be published in front of the class afterwards. The situation was uncontrollable because the number of tasks involved in the intelligence test exceeded all students' capability. During the intervention, students were asked to remain silent to prevent any possible interference by other students. According to one recent study [40] the psychosocial stressor used here produces moderate stress levels according to the cortisol concentration found in a comparable age group. This test can be used in a group setting and is suitable for the use with children. After post-tests of saliva testosterone motor performances were finished and participants were fully debriefed about the real purpose of the study.

### Control condition – normal school stress

In the control group, students took part in a regular teacher-centered biology class for 15 minutes. This represented a situation associated with typical stress experienced by students in school.

### Saliva sampling and analysis

On the day of the experiment, two testosterone saliva samples were taken with Salivette, with the blue cap (Sarstedt, Nümbrecht, Germany). After chewing on a synthetic swab for 1 minute, these swabs were placed in the plastic tube of the salivettes, and collected. Samples were stored at −20 C° until analysis.

The concentration of testosterone in saliva closely correlates with the concentration of free testosterone in blood, and salivary testosterone has been widely confirmed to be a valid and reliable indicator of the biological, active, and free fraction of testosterone levels [42], [43]. Biochemical analyses were performed at the Charité-Universitätsmedizin, Berlin, Germany. Salivary testosterone (pg/ml) was measured by a luminescence immunoassay (IBL, Hamburg, Germany), with a lower limit of sensitivity of 1.8 pg/ml. The intra-assay coefficient of variation was 2.6%, and the corresponding inter-assay coefficient of variation was 4.9%.

### Fine motor skill testing

All groups performed a manual dexterity test (flower trail, FT) from the Movement Assessment Battery for Children-2 (M-ABC-2) pre- and post-intervention to test for changes in fine motor skills [44]. It is a paper pencil test in which the student was asked to follow a curved and angled trail of 46 centimeters with a pencil. Participants were not allowed to change the position of the sheet in front of them and instructed to continuously draw the line. Moreover, students were not allowed to draw over the two parallel lines with 1.5 millimeter space in between that mark the trail. The students had no information about their scores in this task but were able to observe the number of times they crossed the lines and therefore could estimate how their performance was. Each crossing the line was marked as one mistake. Consequently, a high score in the manual dexterity score means more mistakes made. Trained raters evaluated the performances on the M-ABC 2 with a sufficient inter-rater reliability of .92 and an intra-rater reliability of .90.

### Procedure

On the test day, the students refrained from any exercise prior to the investigation. Moreover, students were not allowed to eat two hours before the hormone assessments. The conditions for the exercise group, the psychosocial stress group, and the control group differed only with regard to the stressor. Each participant completed four normal academic school lessons. The first saliva collection took place at the end of the fourth lesson (German, English, or Latin) at 12:00 o'clock to minimize effects of time of day on testosterone levels. Students then received heart rate monitors and accomplished their first FT together in a quiet room. Participants were then separated into the three groups and the exercise intervention took place after walking 50 meters to the gym. The control group remained in one, the psychosocial stress group in another classroom for 15 minutes and participants were not allowed to talk to each other. Also in the exercise group, the subjects were not allowed to talk. After 25 minutes the second saliva collection took place at 12:25 o'clock, followed by the second FT (for all groups together) where the same version of the task was administered. The test ended with the return of the heart rate monitors at 12:45 o'clock. The time lapse was the same for all participants.

### Data analysis

A 2×3 mixed factor analysis of co-variance (ANCOVA) was conducted to test for differences between pre-test and post-test (within), and for differences between the experimental (EG 1 and EG 2) and the control groups (CG) (between). Analyses were conducted separately for T, and manual dexterity performance (FT). Greenhouse-Geisser adjustment was reported when the sphericity assumption was violated. Post-hoc comparisons were conducted to determine pre-to-post-test changes within the three groups (EG 1, EG 2, CG). Analyses have been controlled for gender because manual dexterity and testosterone levels were significantly influenced by gender with boys making more errors in the FT and having higher testosterone levels.

For all analyses, the significance level was α = 0.05. In post-hoc comparisons the nominal alpha level was adjusted following Bonferroni.

## Results

### Descriptive statistics

In Table 1 means and standard deviations can be found for the two dependent variables testosterone and manual dexterity. For both variables pre- and post-test measures are reported. Moreover, separate means (+SD) are given for girls and boys since gender was assumed to have a significant impact on the effects stress exerts on testosterone. At a descriptive level it can be seen that boys show higher pre- and post-stress values for testosterone as well as manual dexterity scores (errors). However, these gender differences were only statistically significant for overall pre-test values of manual dexterity, *t*(60)  = 2.75, *p* = .01, but not for overall post-test values, *t*(60)  = 0.81, *p* = .42. Moreover, for testosterone there were no significant overall gender differences at pre-test, *t*(60)  = 1.83, *p* = .07, and post-test, *t*(60)  = 1.83, *p* = .07. This means that girls made less mistakes in the manual dexterity test before the stress induction. Boys and girls did not differ in fine motor performance after the stressor and showed similar testosterone levels before and after the stressor (see Table 1).

**Table 1:** Means (+SD) for testosterone levels (pg/ml) and manual dexterity scores (mean errors) in response to exercise, psychosocial stress, and in the control group.

	Testosterone	*M* (*SD*)	M-ABC	*M* (*SD*)
	PRE	POST	PRE	POST
**EXERCISE**	**13.09 (8.97)**	**16.90 (12.14)**	**2.10 (2.71)**	**2.90 (3.49)**
Girls	13.71 (11.66)	17.40 (14.75)	1.18 (2.14)	2.18 (3.84)
Boys	12.32 (4.49)	16.30 (8.79)	3.22 (3.03)	3.78 (2.99)
**PSYCHOS. STRESS**	**13.17 (8.43)**	**17.83 (10.46)**	**2.42 (2.99)**	**1.00 (1.29)**
Girls	9.54 (5.36)	15.28 (11.23)	1.67 (2.67)	0.92 (1.24)
Boys	19.39 (9.44)	22.21 (7.87)	3.71 (3.25)	1.14 (1.46)
**CONTROL GROUP**	**10.18 (7.84)**	**13.14 (10.82)**	**1.26 (1.54)**	**0.91 (1.50)**
Girls	7.68 (3.50)	7.67 (4.01)	0.55 (0.52)	1.00 (1.95)
Boys	12.48 (10.00)	18.15 (7.67)	1.92 (2.43)	0.83 (1.03)
**OVERALL**	**12.03 (8.39)**	**15.79 (11.17)**	**1.89 (2.46)**	**1.58 (2.44)**
Girls	10.29 (7.81)	13.51 (11.42)	1.15 (2.02)	1.35 (2.55)
Boys	14.16 (8.72)	18.57 (10.39)	2.79 (2.67)	1.86 (2.32)

### Pre-to-post-test changes of testosterone

A repeated measures ANCOVA was conducted to estimate the effects exercise and psychosocial stress have on testosterone. Gender as a covariate significantly affected students' overall testosterone changes from pre-to-post stress, *F*(1, 58)  = 4.79, *p*<.03, η^2^ = .08. The ANCOVA revealed no significant within-subjects effect for test time, *F*(1,58)  = 3.28, *p* = .08, η^2^ = .05, indicating a different pre-to-post-test testosterone trend for the participants when gender as a covariate was included in the analysis. No significant effect was present for the test by group interaction, *F*(2,58)  = 0.31, *p* = .74, η^2^ = .01. Moreover, no effects between-subjects were found for group allocation, *F*(2,58)  = 1.63, *p* = .21, η^2^ = .05.

### Pre-to-post-test changes of manual dexterity

**Figure 1:**
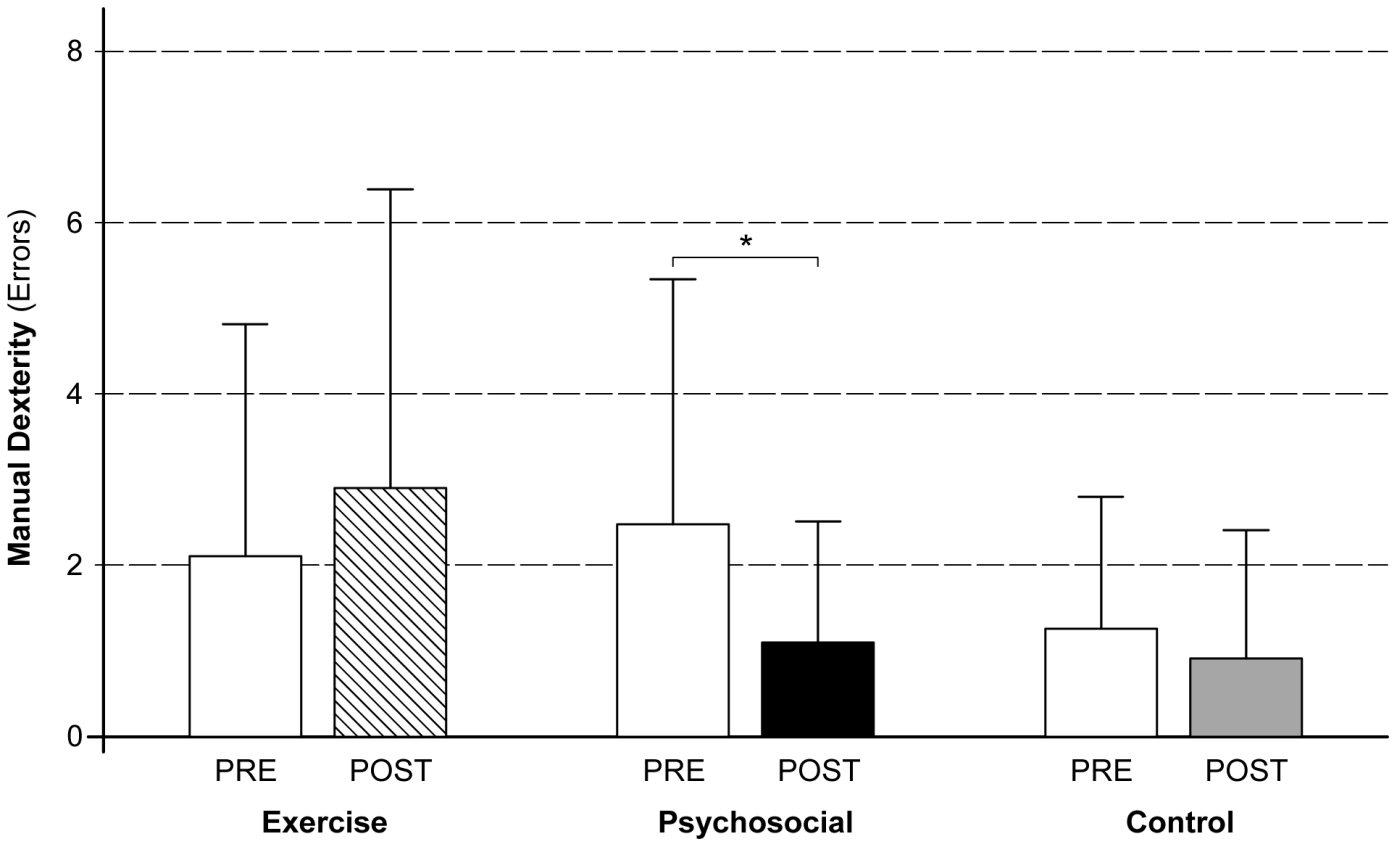
Mean (+SD) errors in the manual dexterity test before and after exercise and psychosocial stress, and for the control group.

The effect of exercise and psychosocial stress on manual dexterity was tested using an ANCOVA. Gender was included as covariate because it significantly affected manual dexterity scores, *F*(1, 58)  = 5.54, *p* = .02, η^2^ = .09. No main effect for test time, *F*(1, 58)  = 0.30, *p*>.58, η^2^ = .01 (within-subjects) could be found. However, the test time by group interaction effect was significant, *F*(2, 58)  = 4.04, *p* = .02, η^2^ = .12, indicating different trends in the manual dexterity performance as effects of the physiological or psychosocial stress. The main between-subjects effect of group allocation was significant. The three groups differed in their average score of manual dexterity, *F*(2, 58)  = 3.28, *p* = .05, η^2^ = .10. Results are illustrated in Figure 1.

#### Post-hoc tests

Within-subjects effects revealed that only participants in the psychosocial stress group significantly improved their manual dexterity from pre to post test (see Figure 1 for illustration), *t*(18)  = 2.65, *p* = .02, d = 0.62, CI (−2.62, −0.22). No within-subjects changes in manual dexterity performance could be shown for the exercise, *t*(19)  = −1.06, *p* = .30, *d* = −0.26, CI (−0.37, 1.97), or the control group, *t*(22)  = 0.84, *p* = .41, *d* = 0.23, CI (−0.74, 1.44). All multiple post-hoc comparisons were Bonferroni corrected.

No significant group differences were present at the pre-test of manual dexterity, *F*(2, 58)  = 2.14, *p* = .13, η^2^ = .07. At post-test, however, participants' manual dexterity scores differed significantly, *F*(2, 58)  = 4.88, *p* = .01, η^2^ = .14. Individual multiple comparisons revealed significantly higher post-test manual dexterity scores, meaning more mistakes, for participants in the exercise group when compared to the psychosocial, *t*(37)  = 2.28, *p* = .03, d = 0.72, CI (0.09, 3.71), and the control group, *t*(41)  = 2.36, *p* = .03, d = 0.74, CI (0.26, 3.72). This means that participants in the exercise group made more mistakes after the stress induction when compared to participants in the psychosocial or control group. Manual dexterity scores in the psychosocial stress group at post-test did not differ from those in the control group, *t*(40)  = 0.20, *p* = .84, d = 0.06, CI (−1.67, 1.84).

### Association between testosterone and manual dexterity

Inter-correlations between pre-, post-, and change-scores (delta) of testosterone and manual dexterity are given in Table 2. As can be seen in Table 2, the overall increase in errors in the manual dexterity task (delta) was related to the overall change in testosterone from pre to post test (*r* = −.31, *p*<.01), meaning that participants showing increases in testosterone also improved their manual dexterity performance, independent of their group allocation. Moreover, the pre-test score of manual dexterity is significantly correlated with the change in testosterone levels (*r* = .27, *p*<.05).

**Table 2:** Pearson correlation coefficients (two-tailed) for pre, post, delta values of testosterone and manual dexterity scores (M-ABC).

	2	3	4	5	6
1 Testosterone (pre)	.72***	−.04	−.02	−.04	−.02
2 Testosterone (post)		.66***	.17	−.08	−.23^†^
3 Δ Testosterone			.27*	−.07	−.31*
4 M-ABC (pre)				.39**	−.56***
5 M-ABC(post)					.55***
6 Δ M-ABC					

Note. ^†^ *p*<0.10, *p<.05, **p<.01, ***p<.001.

## Discussion

The aim of the present study was to investigate how psychosocial and physiological stress affect manual motor performance and whether the different responses are related to different kinetics in the HPG axis expressed through testosterone. Within the present study only psychosocial stress affected manual dexterity, whereas exercise had no effect. By showing that psychosocial stress of 15 minutes led to significant increases of manual precision in the dexterity task we could confirm our hypothesis regarding the effect of psychosocial stress. This finding is in line with Landers and Arent [14], proposing that a moderate increase in arousal would lead to better performance. Apart from this, there are other results showing that psychological stress leads to impaired fine motor, primarily conducted with surgeons [15], [16], or gross motor skills [9], [10], [11], [12]. Still it is difficult to compare the stress levels in the different studies also considering that stress is measured in different ways [16]. The psychosocial stressor used in the present study has been previously shown to increase stress levels moderately [40]. Accordingly, a moderate increase in arousal from base level would lead to a narrowing of attention onto task-relevant cues and, hence, better performance [8], [45].

Our second hypothesis was that motor dexterity would also increase in response to moderate exercise. This theoretical background was also supported by other research groups showing increased motor performances after acute medium intensity exercise when compared to high exercise intensities [6], [8]. On the basis of our data we have to reject this hypothesis. In the present study, students ran with a heart rate of 65–75% of HR_max_, which is slightly below the intensity level in our earlier studies [29], [30]. We could neither find an improvement in manual dexterity nor did we find a testosterone elevation in response to the exercise intensity, which we hypothesized would mediate such a change. One reason could be that the exercise intensity was too low to be able to exert changes to the HPG axis and so was too low to change dexterity. Another possible explanation is that not enough time was given for testosterone levels to actually affect changes at cellular levels in both experimental groups. The structural changes of synapses and their associated dendritic spines after motor training can be modified over a period of minutes to hours [46] and have been shown to be driven by testosterone [47]. A longer time period given after the intervention or for the intervention itself might have altered testosterone levels in a way that also might affect dexterity scores. Future studies might focus on different time intervals given for testosterone to be effective for motor performance.

For testosterone no differences between the experimental groups and no time effect could be found. The descriptive statistics in Table 1 may give a hint on why no significant group differences were present. In both, the exercise and psychosocial stress group individuals showed noticeable increases in testosterone levels from pre- to post-stressor. In the control group no overall testosterone changes were observed. However, only the males in the control increased testosterone levels at a descriptive level when compared to the girls. One could speculate that the performance tests and the general study setting were more stimulating for the boys than for the girls involved. It was previously shown that research involving performance measures raises testosterone levels in male rather than in female participants [48].

A theoretical explanation for altered motor performances in response to psychosocial stress could also be looked for in cognitive psychology. It has previously been proposed that stress – and the activation of negative affect – leads to information processing that focuses on details, and precision [49], [50] for example in recalling memories [51]. The psychosocial stress group in the present study experienced an uncontrollable situation, in which participants were evaluated by others. The situation can be called uncontrollable because the tasks of the intelligence test could not be solved within the time given. This stress reaction then might have led to more focus on detail and precision in working on the manual dexterity task; being more accurate in doing the motor performance.

Importantly, we found that the acute change of testosterone from pre to post test in the present study significantly correlated (*r* = −.31, *p* = .01) with the changes in manual dexterity performances. That means participants showing high acute increases in testosterone from pre to post-test made less mistakes in the fine motor skill task. In a study with elderly men it was previously shown that men with chronically low testosterone levels caused by androgen deprivation therapy experienced worse dexterity scores [25]. In this study, however, researchers looked at chronic changes rather than acute changes in testosterone levels. Thus, the findings by Soyupek et al. [25] cannot be compared with the acute effects in our study. In our present study, we could link acute changes in testosterone to acute changes in fine motor performance. To our knowledge this has not yet been shown previously.

One explanation why testosterone changes may be associated with manual dexterity performance is that testosterone supports the expression of tyrosine hydroxylase (TH) and dopamine transporter (DAT) in the nigrostriatal dopaminergic (NSDA) system which was previously linked to an improvement in motor control [24]. Moreover, Bonifazi et al. [52] suggested that gonadal steroids may modify descending pathways controlling voluntary movements of the hand muscle. Testosterone for example enhances neuronal excitability and facilitates the corticospinal pathways [53].

Additionally, we found a significant positive correlation (*r* = .27) between the pre-score of the motor dexterity performance and the change in testosterone meaning a worse motor performance before the stressor was associated with higher increases in testosterone. From the correlation pattern in Table 2 one might speculate that the pre-test motor dexterity performance had an effect on the change of testosterone. It is well-known that the testosterone levels rise in anticipation of, and during contests [31], [33]. It could be speculated that a student who perceived his performance in the manual dexterity pre-test as insufficient interpreted the test as a contest situation, which consequently led to testosterone increases. However, it appears that a stronger increase in testosterone was associated with improved motor performance in the post-test.

### Limitations, future directions, and practical implications

The results of the present study are subject to some limitations. First, testosterone was only tested pre and post stressor exhibition [54]. Whereas other studies testing effects of stressful situations like competition examined up to eight test times for testosterone [33]. Moreover, we could only correlate testosterone changes with changes in fine motor performance. Future studies should be designed so they can investigate the actual mediation effect of testosterone on students' fine motor performance.

Additionally, we used only one standardized test for manual dexterity in the present study [55]. Results from the present study may be difficult to compare to other tests of motor ability of the hand. Future studies may compare different manual motor tests at the same time.

The present experiment only tested immediate testosterone and manual dexterity changes after acute physiological and psychosocial stressors. From the data at hand, we cannot deduce whether a larger time span between stressor and post-test would benefit manual performance also in the exercise condition. On the basis of our findings and the research literature available future studies should deal with a systematic examination of the delay from onset of the stressor to the manual dexterity testing. In addition, the intensities of exercise but also psychosocial stressors should be systematically controlled to draw conclusions, for example, for school practice. Moreover, the missing analysis of the physical activity status of the participants, which can have an impact on testosterone secretion after acute exercise [36], is a limitation to the present study.

In summary, we found that a moderate psychosocial stressor was significantly able to improve the students' fine motor performance in a school setting. By contrast, moderate exercise of the same duration could not alter manual dexterity performance. Our hypothesis that the kinetics of the HPG axis (measured by testosterone) could be responsible for improved or impaired fine motor skills in response to the two different types of stressors used (exercise vs. psychosocial) had to be rejected. However, overall changes in fine motor performance were significantly correlated with overall changes in salivary testosterone levels independent of the type of stressor induced.
